# Using Real-Time PCR Fluorescence Reaction Values to Improve SARS-CoV-2 Virus Detection and Benefit Clinical Decision-Making

**DOI:** 10.3390/life13030683

**Published:** 2023-03-02

**Authors:** Wan-Wen Yang, Chin-Wen Hsu, Yu-Ju Chan, Shih-Bin Su, I-Jung Feng, Chia-Yi Hou, Chien-Yuan Huang

**Affiliations:** 1 Department of Clinical Pathology, Chi-Mei Medical Center, Liouying, Tainan 736402, Taiwan; 2 Department of Family Medicine, Chi-Mei Medical Center, Liouying, Tainan 736402, Taiwan; 3 Division of Occupational Medicine, Chi-Mei Medical Center, Liouying, Tainan 736402, Taiwan; 4 Division of Occupational Medicine, Chi-Mei Medical Center, Tainan 710402, Taiwan; 5 Institute of Precision Medicine, National Sun Yat-sen University, Kaohsiung 804201, Taiwan

**Keywords:** SARS-CoV-2, relative light unit, clinical decision-making

## Abstract

This study aimed to compare the SARS-CoV-2 nucleic acid detection results of the BD MAX™ System and other platforms to formulate an optimized laboratory verification process. The re-examination of 400 samples determined as positive by BD MAX™ indicated that the inconsistency rate between BD MAX™ and the other platforms was 65.8%; the inconsistency rate of single-gene-positive results was as high as 99.2%. A receiver operating characteristic curve was drawn for the relative light unit (RLU) values of samples positive for a single gene, and RLU 800 was used as the cutoff. After setting the retest standard as single-gene positive and RLU ≥ 800, the number of the 260 BD MAX™ single-gene positives that needed to be confirmed again was 36 (13.8%) and the number that could be directly reported as negative was 224 (86.2%). This verification process can shorten the reporting period and speed up the epidemic adjustment time and turnover rate of special wards, thereby improving SARS-CoV-2 detection efficiency and clinical decision-making.

## 1. Introduction

The outbreak of COVID-19 in December 2019 was declared a public health emergency by the World Health Organization (WHO) on 30 January 2020. Its causative agent is SARS-CoV-2 (severe acute respiratory syndrome coronavirus 2) in the Coronaviridae family of viruses with a single-stranded RNA genome and envelope. SARS-CoV-2 has four structural proteins: spike protein (S), envelope protein (E), nucleocapsid protein (N), and membrane protein (M). The spike protein on the surface of the virus is composed of two domains—S1 subunit (N-terminal) and S2 subunit (C-terminal). The receptor-binding domain (RBD) differentiated from the S1 subunit binds to the angiotensin-converting enzyme 2 (ACE2) receptor on the surface of the host cell to further infect the cell, which becomes the key to viral infection in humans [1]. Transmission routes include respiratory droplets, direct contact with an infected person, short-range aerosols, the touching of virus-contaminated objects, and airborne aerosols [2]. After viral infection, tissue damage occurs, which in turn causes the immune system to respond, resulting in a large secretion of cytokines. Severe immune system imbalance will produce an excess of the proinflammatory cytokines IL-6 and TNFα, resulting in “cytokine storm” systemic inflammation [3]. After diagnosis, most people infected with SARS-CoV-2 have mild or no symptoms. Common clinical manifestations of COVID-19 include fever, fatigue, and dry cough. Other less common symptoms include headache, runny nose, sore throat, diarrhea, shortness of breath, and an abnormal sense of smell or taste. A small number of infected people progress to severe pneumonia, respiratory distress syndrome, multiple organ failure, shock, and even death [4,5]. Chakraborty et al. suggest that IL-6 antagonists can be used to treat the cytokine storm caused by SARS-CoV-2 [6]. According to the WHO, the total number of confirmed cases from the initial outbreak up to August 2022 was 588,757,628, with a mortality rate of about 10.9%. Since SARS-CoV-2 is an RNA virus, its genes are prone to mutation, which leads to the continual appearance of mutant variants of the virus, such as Alpha (B.1.1.7), Beta (B.1.351), Delta (B.1.617.2), Gamma (P.1), and Omicron (B.1.1.529), and new genotypes are still being discovered in various countries [7]. Despite SARS-CoV-2 pandemic prevention strategy planning, the large number of asymptomatic carriers makes it extremely difficult to control the infection and clinical testing a major challenge, thereby increasing the public health burden [8,9].

Taiwan’s population density is high [10]. Therefore, speeding up diagnosis and epidemic investigation have become the most important goals of hospitals and inspection agencies. Detection of SARS-CoV-2 RNA is the gold standard for diagnosis. Hospitals have established relevant inspection procedures and introduced equipment for detecting nucleic acids. Three platforms are used for detecting the virus—Cepheid Xpert Xpress (hereinafter referred to as Xpert), BD MAX™, and LabTurbo™ AIO (hereinafter referred to as AIO)—to cope with the large number of SARS-CoV-2 tests performed in the country during the epidemic. Although the methodology of each instrument is real-time PCR, due to differences in the design of reagents used in the systems, test performance is also different. For example, BD MAX™ contains high-affinity magnetic beads for capturing viral nucleic acids [11]; the Xpert SARS-CoV-2 test combines all reagents and samples in a disposable cassette for the reaction [12]; AIO uses membrane tube vacuum flow technology to extract high-purity RNA for the reaction [13]. In addition to these differences, the machines also differ in the way they detect target gene fragments and in their detection limits. When the reports are integrated and interpreted according to these differences, the detection of two genes at the same time is regarded as a positive result. If a single gene is positive or the Ct value is >38 and the viral load is low, the Centers for Disease Control recommend retesting or resampling to improve the accuracy of the report. However, retesting or resampling will delay reporting, which will affect the business process of the clinical operation.

**Figure 1:**
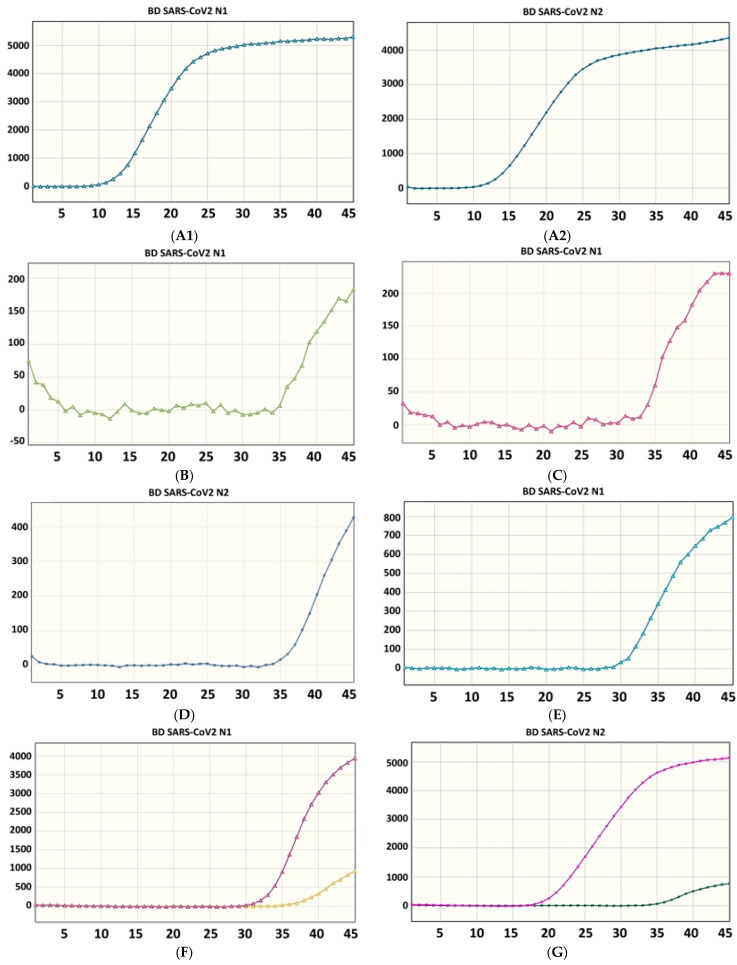
Ct values to help determine true positive and false positive results obtained from the BD MAX™ SARS-CoV-2 platform. (**A1**,**A2**) double-gene (N1 and N2) sigmoidal curve for true positives. (**B**,**C**) single-gene (N1) non-sigmoidal curves for false positives. (**D**,**E**) single-gene (N1 or N2) threshold for false positives over 400 curves. (**F**) N1 curve for true positives and false positives in the same batch. (**G**) N2 curve for true positives and false positives in the same batch.

In addition, because of the continual adjustment of infection control policies by the Epidemic Command Center and the increased rate of vaccine administration, in addition to SARS-CoV-2 RNA testing as the diagnostic standard, nucleic acid test reports are also required for the admission of quarantined patients to special wards. According to the conditions for isolation and treatment of severe COVID-19 cases, revised by the Command Center on 31 May 2022, if a confirmed case’s fever has gone for at least one day and the symptoms are relieved, the following conditions should be met for release from isolation following evaluation by a doctor: the PCR test result of one respiratory specimen is negative or the Ct value ≥ 30; the date of onset or sample collection has reached 15 days; and the Ct value of the PCR of a respiratory specimen is between 27 and 30. Therefore, the establishment of an effective mechanism for interpretation of SARS-CoV-2 reports in the laboratory is highly important for the clinic. Previous studies have shown that BD MAX™, compared to Xpert results, can have a false positive rate of about 3.5% [14,15]. However, the Ct graph can be viewed on the BD MAX™ instrument (Figure 1) to help further confirm the result [14]. When the N1 or N2 gene is positive and the results for a large number of samples need to be reported urgently, the rapid and reasonable cross-interpretation of the classical sigmoidal curves and fluorescence values is undoubtedly a significant challenge for medical examiners.

According to data from the Taiwan Centers for Disease Control, the nationwide rate of confirmed cases as of 31 March 2022, was 0.78%; thus, the Central Epidemic Command Center announced that a 1:10 pooled PCR is required for specific groups. However, an outbreak began that raised the nationwide rate of confirmed cases to 25.99% in April 2022, alongside the positivity rate of 1:10 pooled PCR results. Nevertheless, due to the rapid course of the epidemic, the operating procedures were not adjusted accordingly, resulting in a large increase in the rate of positive pooled samples after changing to single PCR operation. This not only delays reporting but also increases the testing costs. This also includes retests for confirming whether the positive single-gene result of the BD MAX™ SARS-CoV-2 pooled sample is a false positive. It is extremely important to issue accurate reports, but repeated testing of samples to confirm results will result in a reduction in the number of tests, as well as affecting the epidemic investigation time and the turnover rate of special wards, and might even lead to the situation of no vacant beds for patients who need to be hospitalized. Therefore, we should seek a balance between the two.

In the past, SARS-CoV-2 retesting was performed to reduce false-positive results, which increased laboratory workload and reporting time. Therefore, SARS-CoV-2 retest criteria were developed in a previous study which set an initial relative light unit (RLU) value of 1000 as the cutoff for retesting; when RLU ≥ 1000, 98% of the test results were confirmed as positive, whereas when RLU < 1000, only 32% were confirmed as positive [16]. Another study analyzed the results of BD MAX™ and Xpert. When the Ct value was >34, 92% of the results were inconsistent, and it was often necessary to re-collect samples to confirm the correctness of the results [14]. Therefore, in this study, we compared the detection results of the BD MAX™ System with those of the other two platforms. We analyzed the parameter characteristics of BD MAX™ and sorted them to formulate an optimized laboratory verification process that complies with the national quarantine policy.

## 2. Materials and Methods

### 2.1. Data Collection

This study was approved by Institutional Review Board, Chi-Mei Medical Center, Liouying (IRB No.:11111-L02). Between 23 May 2021 and 8 May 2022, nucleic acid testing of routine nasopharyngeal samples was performed to detect SARS-CoV-2 by the laboratory of Chi-Mei Medical Center, Liouying, Tainan, Taiwan. The BD MAX™ System obtained positive results for 400 samples.

### 2.2. Equipment

Three platforms were used for detecting SARS-CoV-2 nucleic acid: BD MAX™ (MAX SARS-CoV-2, Becton, Dickinson and Company; BD Life Sciences—Integrated Diagnostic Solutions, Sparks, MD, USA); Gene Xpert System (Cepheid, Sunnyvale, CA, USA); and LabTurbo™ AIO48 (LabTurbo, Taipei, Taiwan). The target genes of the BD MAX™ SARS-CoV-2 reagent were N1, N2, and Ribonuclease P (RNaseP); the sample volume was 750 μL; the limit of detection (LOD) was 5400 NDU/mL; and the turnaround time was about 2.75 h/24 samples. The target genes of Xpert SARS-CoV-2 reagent were E and N2; the sample volume was 300 μL; the LOD was 5400 NDU/mL; and the turnaround time was 45 min per sample. In the case of AIO COVID-19 RNA reagent (Acov11240), the target genes were E, N1, and RNaseP, with a LOD of 2 copies/μL, 1 copy/μL, and 0.1 copy/μL, respectively; the sample volume was 500 μL, and turnaround time was 2.5 h/48 samples. We used the primers and probe sequences provided by three different real-time PCR reagents to detect the target genes (E, N1, and N2) of SARS-CoV-2 in the samples (Table 1).

### 2.3. RNA Extraction

#### 2.3.1. BD MAX™ SARS-CoV-2 Assay

The BD MAX™ ExK™ TNA-3 unitized reagent strip contains a combination of lytic and extraction reagents designed to perform cell lysis and total nucleic acid (TNA) extraction. Nucleic acids released from the target organisms are captured on magnetic affinity beads. The beads, together with the bound nucleic acids, are washed and the nucleic acids are eluted by a combination of heat and pH variation.

#### 2.3.2. Cepheid Xpert^®^ Xpress SARS-CoV-2 Assay

The Cepheid Xpert^®^ Xpress SARS-CoV-2 test is performed on the Gene Xpert system that automates and integrates sample preparation, nucleic acid extraction, amplification, and detection of the target sequences using real-time RT-PCR assays. This system uses single-use disposable cartridges that hold the RT-PCR reagents and host the RT-PCR process.

#### 2.3.3. LabTurbo AIO COVID-19 RNA

LabTurbo Kits use silica membrane-chaotropic technology for the purification of high-quality RNA from samples. The nucleic acid extraction process includes decomposing the virus particles in the sample, binding the virus nucleic acid to the tube column membrane, cleaning the silicon membrane, and recovering the virus nucleic acid from the membrane.

### 2.4. Interpretation of Patient Specimen Results

#### 2.4.1. BD MAX™ SARS-CoV-2 Assay

The BD MAX™ system has an software for the interpretation of results with an established threshold for calling positives or negatives. The presence of N1 or N2 genes is reported as a positive result. Negative N1 and N2 results alongside positive RNase P results are interpreted as not detected. Unresolved N1 or N2 results with unresolved RNase P results are interpreted as unresolved (UNR) and require repeat testing. Positive results are indicative of the presence of SARS-CoV-2 RNA [14].

#### 2.4.2. Cepheid Xpert^®^ Xpress SARS-CoV-2 Assay

The results were interpreted according to the manufacturer’s instructions. If both targets are detected or if only target N2 is detected, the test reports a positive result. If only target E is detected, the test reports a presumptive positive result because this target is shared by some members of the Sarbecovirus subgenus of coronaviruses; in this case, the test must be repeated. The Xpert Xpress SARS-CoV-2 test generates a positive result when a signal for the N2 region or signals for both nucleic acid targets (N2 and E) have a Ct within the valid range (<45 Ct) and an endpoint above the minimum setting [20].

#### 2.4.3. LabTurbo AIO COVID-19 RNA

A FAM signal indicates that the N gene of SARS-CoV-2 (COVID-19) has been detected; a HEX signal indicates that the E gene of universal coronavirus has been detected. COVID-19 RNA is detected when a signal for the N1 region or signals for both nucleic acid targets (N1 and E) have a Ct within the valid range (<36 Ct) and an endpoint above the minimum setting. A presumptive positive result is reported when the SARS-CoV-2 signal for only the E nucleic acid target has been detected.

### 2.5. Data Analysis

The 400 samples that were determined as positive by the BD MAX™ System were randomly tested on other platforms (Xpert or AIO) for repeated confirmation within 24 h, and the results were compared retrospectively. If both platforms generated positive results for both genes, the specimen was deemed positive, and if only a single platform produced a positive result, then the specimen was deemed negative. The best cutoff point was analyzed with R software (version 4.2.1).

## 3. Results

The BD MAX™ SARS-CoV-2 test identified 400 samples as positive. These 400 samples were randomly re-examined on either the Xpert or the AIO platform; the re-examination resulted in 137 positive samples and 263 negative samples. These results indicated that the rate of inconsistency between the 400 positive results of the BD MAX™ SARS-CoV-2 assay and the retest results was 65.8%.

**Figure 2:**
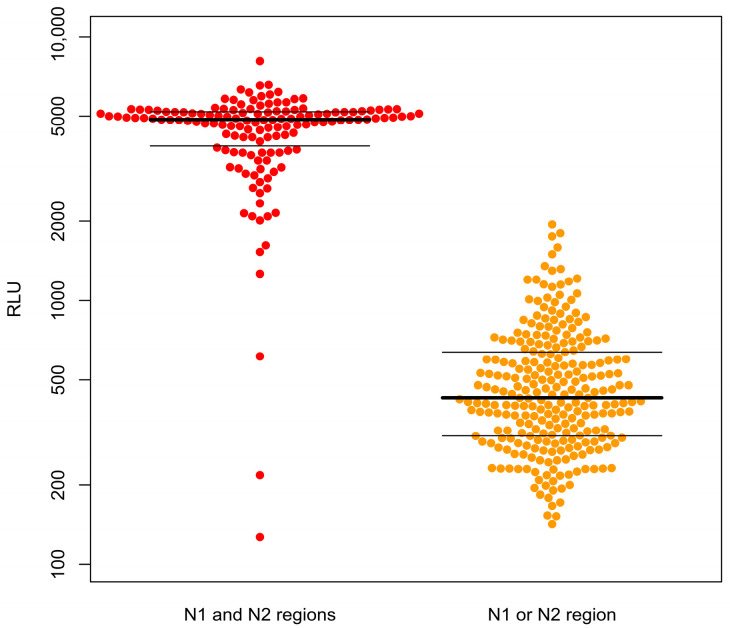
Distribution of RLU values of samples tested using the BD MAX™ SARS-CoV-2 platform, which were positive for the N1 or N2 region (single gene) or both N1 and N2 regions (double gene).

BD MAX™ further differentiated the results by gene expression: 135 of the double-gene (N1 and N2 regions)-positive samples were positive after retesting, and five were negative; two of the single-gene (N1 or N2 region)-positive samples were positive after retesting, while 258 were negative. These results indicate that samples positive for a single gene (N1 or N2 region) in the BD MAX™ test have a high rate of inconsistency in retests, with 99.2% showing negative results (Table 2). We further analyzed the relevant parameters of these specimens and found significantly different distributions of RLU in the single-gene-positive and double-gene-positive groups. The median RLU was 4852 for the double-gene positive group and 427 for the single-gene positive group (Figure 2).

**Figure 3:**
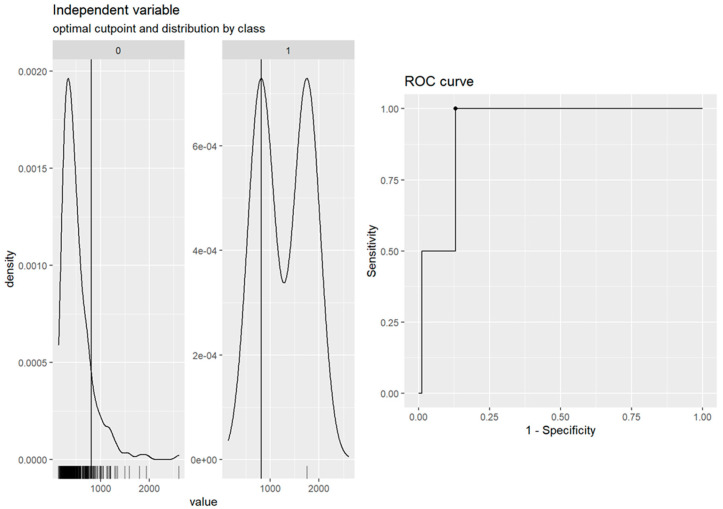
The receiver operating characteristic (ROC) curve drawn with the RLU values and recheck values of samples testing positive for the N1 or N2 region (single gene) in the BD MAX™ SARS-CoV-2 platform.

Therefore, we plotted a receiver operating characteristic (ROC) curve based on the RLU values and retest results of the single-gene-positive samples of the BD MAX™ SARS-CoV-2 assay. The area under curve (AUC) was 0.93, which confirmed that RLU 813 can be used as the cutoff value for the BD MAX™ SARS-CoV-2 assay results of single-gene-positive low false positives. Due to laboratory threshold setting and rounding, RLU 800 was finally used as the adjusted cutoff value (AUC = 0.87) (Figure 3).

The above results show that among the 260 single-gene positives from BD MAX™, there were 36 cases with RLU ≥ 800, of which 34 were negative on re-examination and two were positive, and there were 224 cases with RLU < 800, all of which were negative on re-examination. The 86.8% of the single-gene-positive cases from the BD MAX™ SARS-CoV-2 testing that had an RLU of < 800 can be directly deemed negative without retesting. The cutoff of RLU 800 has a sensitivity of 100% and specificity of 86.8% (Table 3).

## 4. Discussion

**Figure 4:**
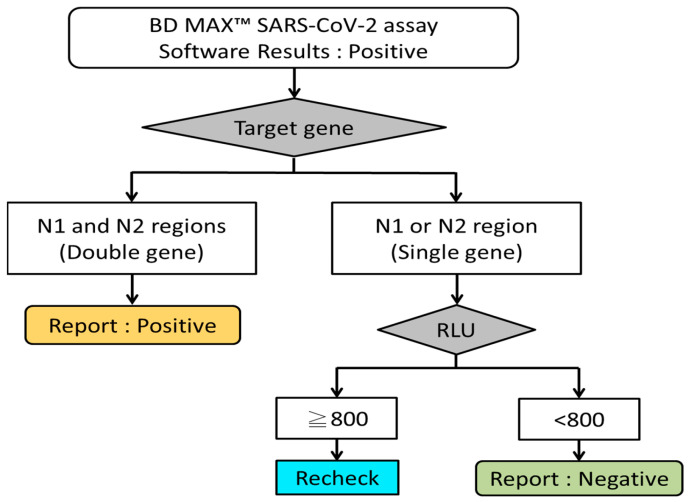
The validation and reinspection process for positive results obtained with the BD MAX™ SARS-CoV-2 platform.

**Figure 5:**
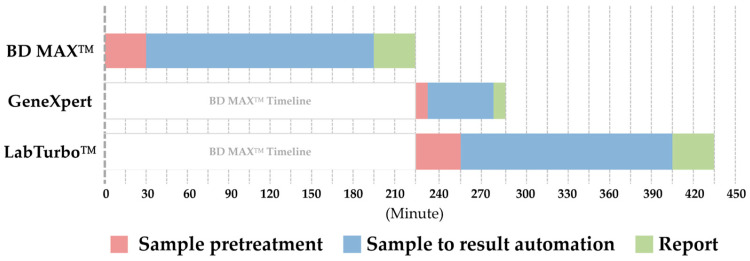
The time required for BD MAX™ SARS-CoV-2 testing and the overall time spent on retesting.

In this study, the ROC curve was drawn based on the RLU and retest results of the single-gene-positive samples from the BD MAX™ SARS-CoV-2 assay; the best cutoff value of RLU was 813 (AUC = 0.93). Due to laboratory threshold setting and integer rounding, the cutoff value was finally adjusted to RLU 800 (AUC = 0.87). Although the cutoff of RLU = 800 will increase the proportion of laboratory retests, given that there may be omissions near the critical value, we chose it as the adjusted cutoff rather than a higher cutoff to avoid true positives being excluded. The comparison of results from the three platforms showed that on the BD MAX™ platform, if the retest standard was set as single-gene positive and an RLU of 800, the proportion of reconfirmations (RLU ≥ 800) was 36/260 (13.8%), while the proportion of direct negative reports (RLU < 800) was 224/260 (86.2%) (Figure 4). The overall time required on the BD MAX™ platform was approximately 225 min. Without considering whether the instrument was fully loaded and whether it could be used immediately, the overall time required for re-examination was about 280 min with the Xpert platform, but about 435 min with the AIO platform (Figure 5). Therefore, the retest standard not only saves reagent costs for 224 retests, but also reduces the time spent on retesting (about 1–3.5 h) and issuing reports, and thus improves the laboratory-side SARS-CoV-2 detection efficiency. AlBahrani et al. reported that SARS-CoV-2 Ct values do not show any association with clinical symptoms and do not predict the need for mechanical intubation or death [21]. Another study showed that gender, age, co-morbidity, and mortality do not differ significantly between patients with low (≤25) and those with high (>25) Ct values [22]. However, in Coyle et al.’s study, Ct ≤ 30 was reported as positive and Ct > 30 as reactive and interpretive comments were added to all reports; these reports had an impact on patient and public health management criteria [23]. It is important to provide a critical result for clinical purposes.

The pooling of specimens for testing has been shown to be an effective approach to expand detection capacity, reduce per specimen costs, and shorten turnaround time (TAT). However, false-negative results are more likely due to specimen dilution after pooling [24,25]. Watkins et al. suggested that the pooling ratio can be evaluated using the community positivity rate. If the prevalence is greater than 3%, a pooling ratio of 1:5 is recommended. However, if the prevalence is less than 1%, the pooling ratio can be 1:10–1:20 to improve the efficiency of screening operations. This study suggested that individuals perform the tests twice a week, and the false negative rate was around 1.96% [26]. In April 2022, the Central Epidemic Command Center recommended a 1:10 pooled PCR operation. A previous study showed a positive percent agreement (PPA) for the combined test versus the individual test ranging from 71.7% to 82.6% for an 8-person group and from 82.9% to 100.0% for a 4-person group [27]. False-negative results occurred exclusively in pools containing samples with low estimates of viral load (Ct > 34) [27]. Handous et al. found that the sensitivities of pooling ratios 1:5 and 1:10 were not significantly different (*p* > 0.05); however, the pooled testing Ct values exceeded those of individual samples in 5-sample and 10-sample tests, being 1.85 ± 1.09 and 3.4 ± 1.65 cycles, respectively [28]. Another study mentioned that the probability of 10-sample pooled tests with a false- negative result (13.3%) if the CT values of specimens were low (>35) [29]. Another related study showed that concordance was ≥90% across all laboratories for 5-sample pools [30]. Healy et al. mentioned that the low prevalence ratio should consider the false positive if the CT values were low [31]. Some procedures in PCR should be prevented such as environmental swab contamination and positive located near to a stronger positive [32]. After synthesizing the above research results, evaluating COVID-19 prevalence in southern Taiwan, and verifying the instrument’s test limit value, our laboratory uses 1:5 as the sample pooling operation specification for the BD MAX™ platform. Then, the individuals, especially high risk medical staff and inpatients, perform the test once a week, following the policy of the Centers for Disease Control.

Diagnosis of SARS-CoV-2 is primarily based on RT-PCR analysis that amplifies at least two viral target genes, but in some cases only one gene. Falasca et al. suggested that it is easy to produce false positives when the viral load is low; when Ct > 39 and the samples are single-gene positive, the retest result is negative, but when the single-gene Ct is around 38 and there are clinical symptoms such as cough and fever, the viral culture is positive [20]. Fenaux et al. analyzed test results from March to August 2020, and found that one target positive (OPT) results accounted for 11% of positive RT-PCR results and 1.5% of all RT-PCR results; 68% of patients with OPT results were classified as likely to have COVID-19, mainly because their previous RT-PCR was positive for dual target genes and they were likely to be at an advanced stage of infection with low viral load. Conversely, OPT results may also reflect viral replication starting before symptoms appear, in which case contagiousness would be high after a few days [33]. In this study, two cases were single-gene positive in the BD MAX™ SARS-CoV-2 test and their retest results were also positive; their Ct values were >34. One of the samples was a nasopharyngeal specimen collected from the patient during isolation and hospitalization, and its RLU value was 813, which indicated an advanced stage of infection. The other sample was a nasopharyngeal specimen from a patient who developed fever, upper respiratory tract symptoms, abdominal pain, diarrhea, and chest pain three days before the test. The RLU value was 1752, which indicated an early stage of infection. Although a Ct value of > 34 indicated a state of low viral load, the clinical conditions of the two cases were completely different. Therefore, in the initial stage of infection, when the viral load is low and clinical symptoms appear, according to the conclusions of this study, a single-gene positive result with an RLU > 800 needs to be retested and should not be excluded.

Few limitations of our study require consideration. The laboratory was not equipped with three platforms prior to the outbreak. Therefore, we were unable to obtain the confirmatory results of a single sample on all three platforms. Negative samples were not retested due to the high testing loads during the peak of the local epidemic. The ROC curve in this paper is drawn based on the positive RLU and retest results of a single gene in BD MAX™ SARS-CoV-2 assay, and the number of analyzed samples is not enough (n = 260). Therefore, for the cut-off value determined for single-gene positive, it is necessary to increase the number of samples in the future to improve the reliability. Second, this study only collected data from tests performed in the Chi-Mei Medical Center, Liouying, Tainan, Taiwan, so it was impossible to confirm the course of the patient’s infection and medication. Finally, because this article only focuses on the detection of SARS-CoV-2 on the BD MAX™ platform, whether the research results are applicable to all real-time PCR platforms depends on the results of future research on other platforms.

## 5. Conclusions

Every laboratory creates its own confirmatory testing pathways based on its own capacity, pressures, and resources. In this study, the retest standard was BD MAX™ SARS-CoV-2 single-gene positivity with the reference RLU value. The retest standard not only saves reagent costs incurred in repeated testing, but also reduces the time spent on retesting and reporting, and accelerates the epidemic adjustment time and turnover rate of dedicated wards, thereby improving the efficiency of SARS-CoV-2 detection and clinical decision-making.

## Figures and Tables

**Table 1:** The primers and probe sequences used with three different real-time PCR reagents to detect SARS-CoV-2.

Equipment	Target Gene	Primers and Probe	Oligonucleotide Sequence (5′ to 3′)	Reference
BD MAX™	N1	Forward primer	GACCCCAAAATCAGCGAAAT	[17]
		Reverse primer	TCTGGTTACTGCCAGTTGAATCTG	
		Probe	FAM–ACCCCGCATTACGTTTGGTGGACC–BHQ1	
	N2	Forward primer	TTACAAACATTGGCCGCAAA	
		Reverse primer	GCGCGACATTCCGAAGAA	
		Probe	FAM–ACAATTTGCCCCCAGCGCTTCAG–BHQ1	
	RNase P	Forward primer	AGATTTGGACCTGCGAGCG	
		Reverse primer	GAGCGGCTGTCTCCACAAGT	
		Probe	FAM–TTCTGACCTGAAGGCTCTGCGCG–BHQ1	
LabTurbo™	E	Forward primer	ACAGGTACGTTAATAGTTAATAGCGT	[18]
		Reverse primer	ATATTGCAGCAGTACGCACACA	
		Probe	FAM-ACACTAGCCATCCTTACTGCGCTTCG-BBQ	
	N1	Forward primer	GACCCCAAAATCAGCGAAAT	
		Reverse primer	TCTGGTTACTGCCAGTTGAATCTG	
		Probe	/56FAM/ACCCCGCAT/ZEN/TACGTTTGGTGGACC/3IABkFQ/FAM/ACCCCGCAT/ZEN/TACGTTTGGTGGACC/3IABkFQ/	
GeneXpert	RNase P	Forward primer	AGATTTGGACCTGCGAGCG	[19]
		Reverse primer	GAGCGGCTGTCTCCACAAGT	
		Probe	/5Cy5/TTCTGACCT/TAO/GAAGGCTCTGCGCG/3IAbRQSp/	
	N2	Forward primer	TTACAAACATTGGCCGCAAA	
		Reverse primer	CGCGCTGTAAGGCTTCTT	
		Probe	CACAATTTGCCCCCAGCGCTTC	
	E	Forward primer	Confidential issue	
		Reverse primer	Confidential issue	
		Probe	Confidential issue	

**Table 2:** Comparison of retest results from the BD MAX™ SARS-CoV-2 platform according to gene expression.

	Confirmed Negative	Confirmed Positive	Total
N1 and N2 regions	5	135	140
N1 or N2 region	258	2	260
Total	263	137	400

**Table 3:** Combined results for the retest of samples that were initially tested with the BD MAX™ SARS-CoV-2 platform and were positive for the N1 or N2 region (single gene). RLU 800 was the cut-off value.

Gene	RLU	Confirmed Negative	Confirmed Positive	Total
N1 or N2 region	RLU ≥ 800	34	2	36
	RLU < 800	224	0	224
Total		258	2	260

## Data Availability

All data are fully available without any restriction upon reasonable request.
